# Supplementary material for: Epstein–Barr Virus BRRF1 Induces Butyrophilin 2A1 in Nasopharyngeal Carcinoma NPC43 Cells via the IL-22/JAK3-STAT3 Pathway
Source: Int J Mol Sci. 2024 Dec 15;25(24):13452. doi: 10.3390/ijms252413452 (PMC11677325; doi:10.3390/ijms252413452)
Supplement: Supplementary file 1 [file ijms-25-13452-s001.zip › ijms-3353253-supplementary.pdf]

Figure S1

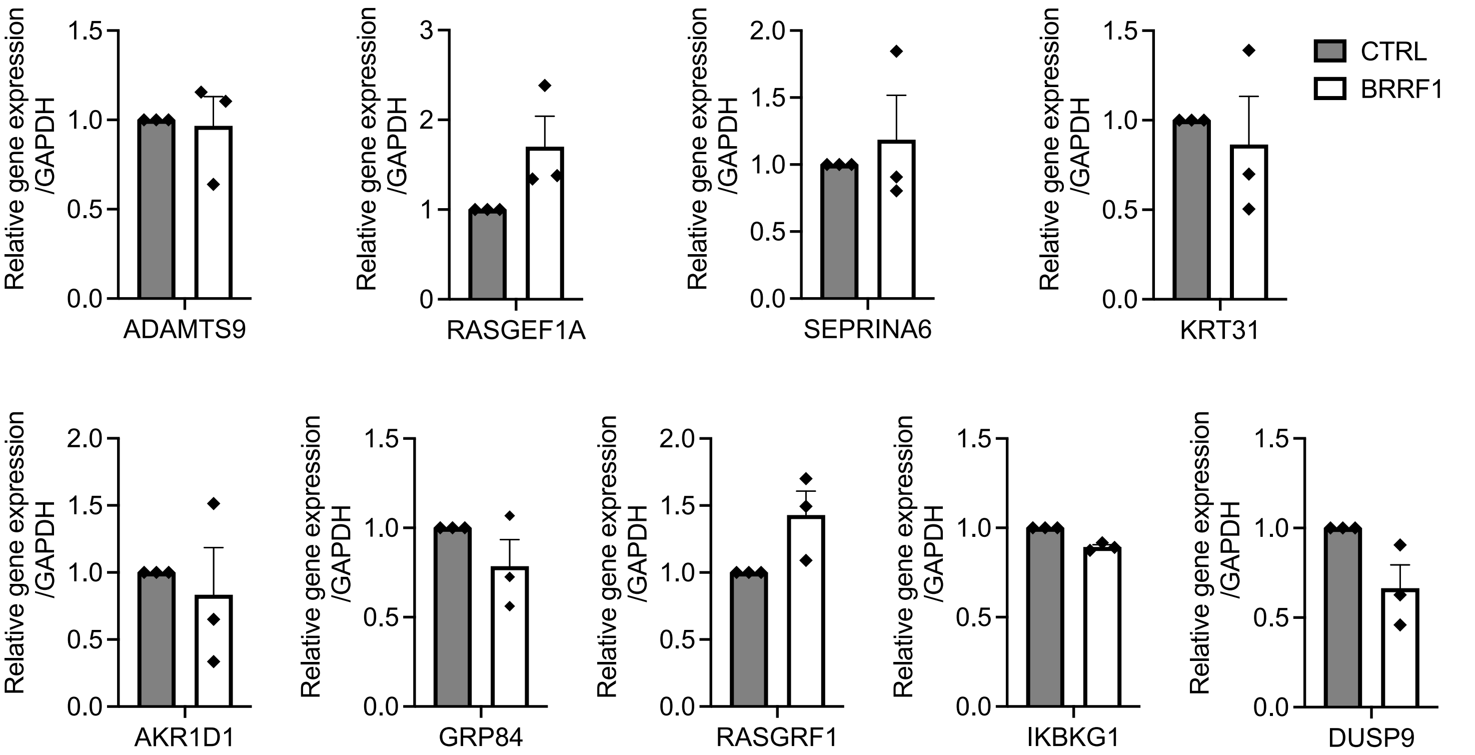

Figure S1. Up-regulated gene mRNA expression was measured in BRRF1 overexpressed NPC43 cells by RT-qPCR. *ADAMTS9*, *RASGEF1A*, *SEPRINA6*, *KRT31, AKR1D1*, *GRP84*, *RASGRF1*, *IKBKG1,* and *DUSP9* gene mRNA expression was measured by RT-qPCR normalized to control (CTRL).

Figure S2

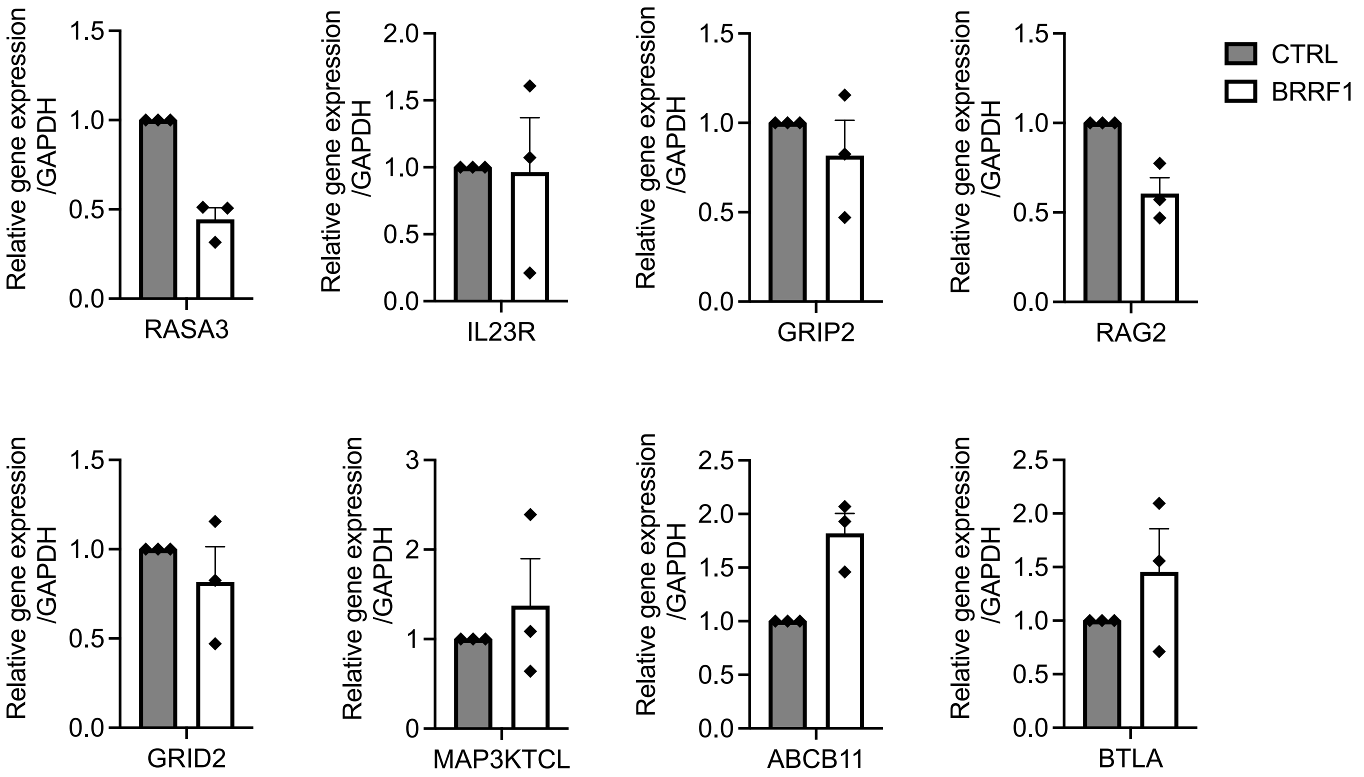

Figure S2. Down-regulated gene mRNA expression was measured in BRRF1 overexpressed NPC43 cells by RT-qPCR. *RASA3*, *IL23R*, *GRIP2*, *RAG2*, *GRID2*, *MAP3KTCL, ABCB11*, *BTLA* gene mRNA expression was measured by RT-qPCR normalized to control (CTRL).

.

Figure S3

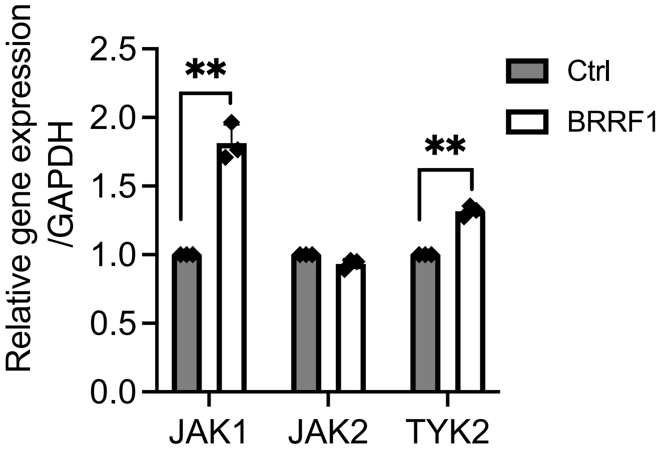

Figure S3. NPC43 cells were transfected with BRRF1 for 3 h and assessed for *JAK1*, *JAK2*, and *TYK2* gene expression by RT-qPCR. Data represents mean ± SEM from 3 independent experiments. Student's *t* test was performed for statistical analysis. $^{**}P < 0.01$.

Figure S4

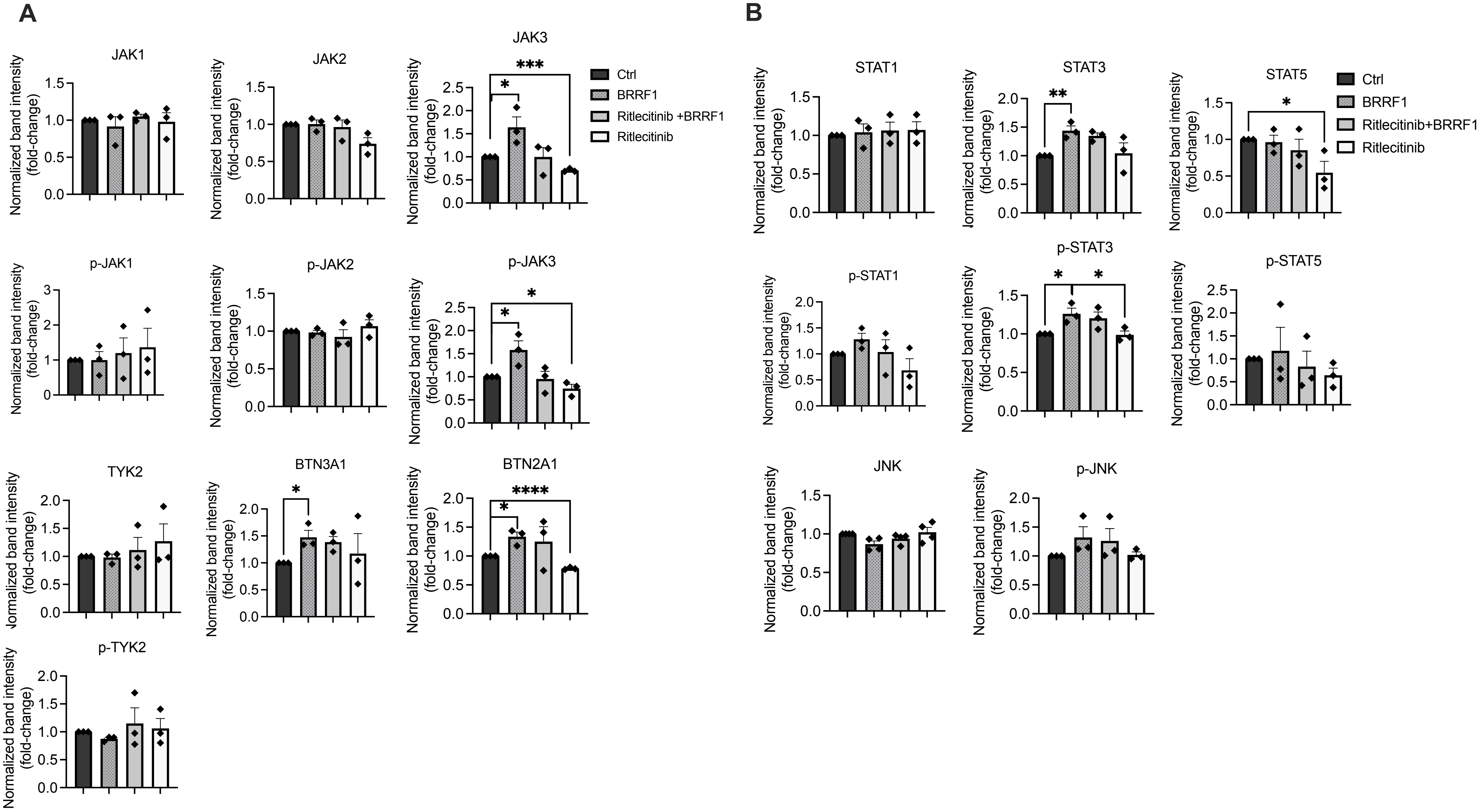

Figure S4. Western blot analysis of different JAK and STAT pathway protein expression of NPC43 cells with different treatment groups measured by band intensities. (A) JAK family proteins, BTN2A1 and BTN3A1, and (B) STAT family proteins relative band intensities normalized to β-actin are shown. Data from 3 independent experiments are shown as mean ± SEM in the column graphs. Student's *t*-test was performed. *$P < 0.05$, **$P < 0.01$, ***$P < 0.001$, ****$P < 0.0001$.

Figure S5

A NPC43

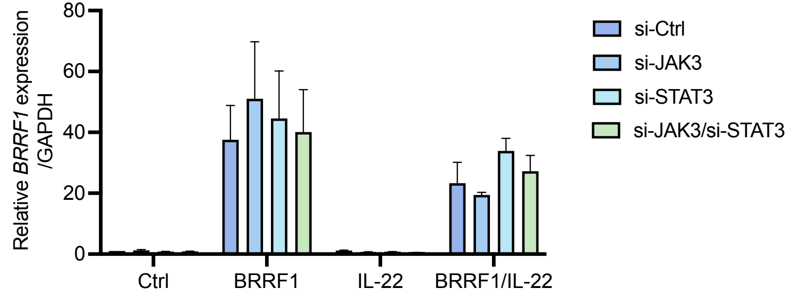

B HK1-EBV

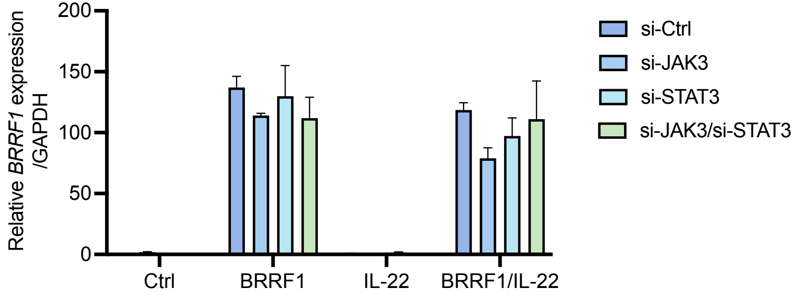

Figure S5. Overexpression of *BRRF1* following transfection in NPC43 or HK1-EBV cells. Detection of *BRRF1* expression at 24 h after transfection in (A) NPC43 and (B) HK1-EBV cells treated for 48 h with siRNA against JAK3 and/or STAT3, or scrambled control. Data from 3 independent experiments are shown as mean ± SEM in the column graphs.

Figure S6

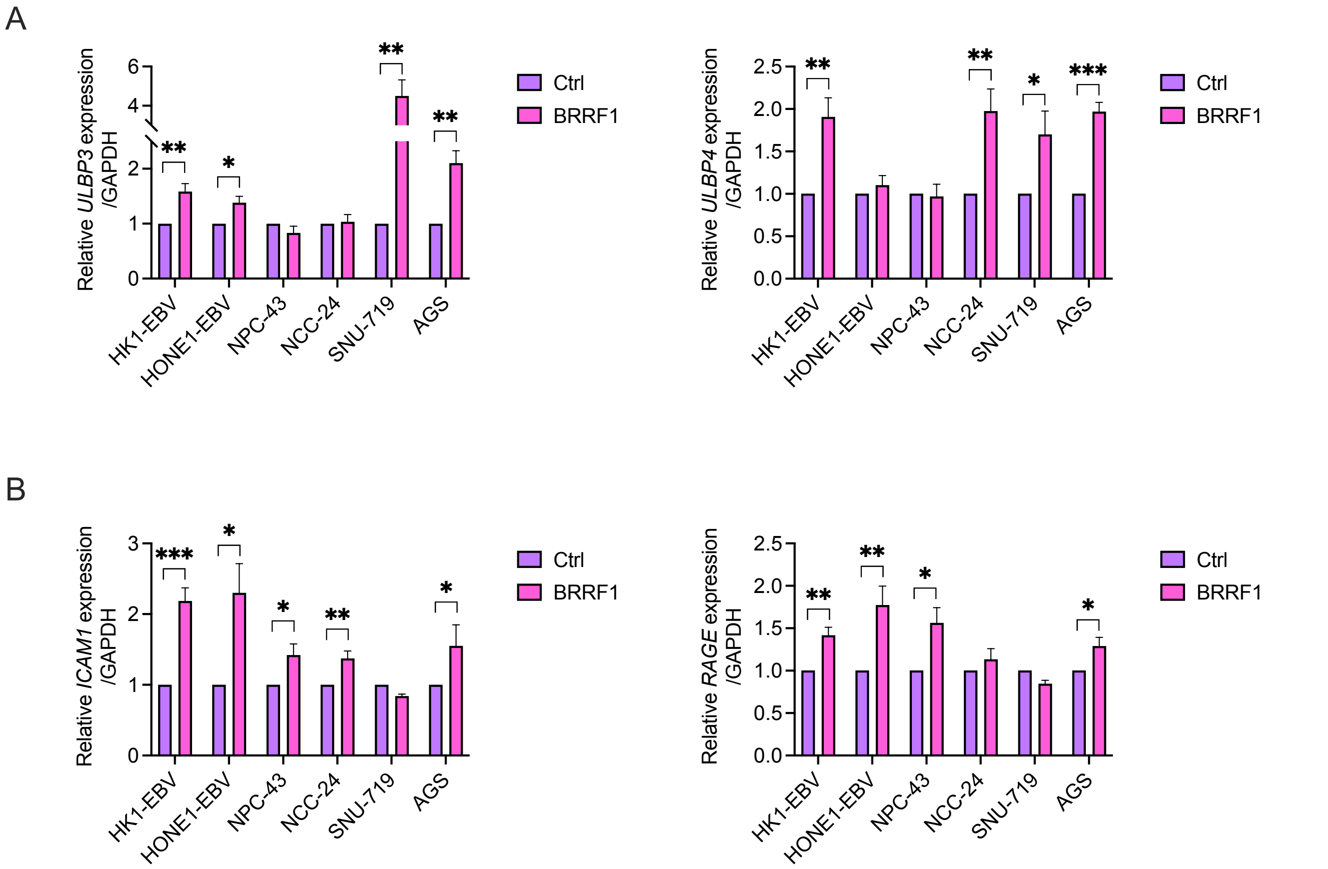

Figure S6. BRRF1 overexpression upregulates NKG2D ligands and adhesion molecules in tumor cell lines. Cells were transfected with BRRF1 and assessed for the gene expression of (A) NKG2D ligands *ULBP3* and *ULBP4* expression, or (B) adhesion molecules *ICAM1* and *RAGE*, by RT-qPCR. Data shown as the mean ± SEM from 3 independent experiments. Student's *t*-test was performed for statistical analysis. *$P < 0.05$, **$P < 0.01$, ***$P < 0.001$.

Table S1. Selected up-regulated genes and its function in BRRF1 -overexpressed NPC43 cells.

Gene	Gene description	Gene function
RASGEF1A	RasGEF domain family member 1A	Positive regulation of Ras protein signal transduction
JAK3	Janus kinase 3	Cytokine receptor-mediated intracellular signal transduction
AKR1D1	Aldo-keto reductase family 1 member D1	Responsible for Synthesis of bile acids and bile salts and Metabolism.
KRT31	Keratin 31	Keratinization and Nervous system development
SERPINA6	Serpin family A member 6	Major transport protein for glucorticoids and progestins in the blood of most vertebrates
GPR84	G protein-coupled receptor 84	Involved in neuropeptide signaling pathway
IKBKGP1	Inhibitor of nuclear factor kappa B kinase subunit gamma pseudogene 1	Pseudogene
DUSP9	Dual specificity phosphatase 9	Negatively regulate members of the MAP superfamily (MAPK/ERK, SAPK/JNK, p38), associated with cellular proliferation and differentiation
RASGRF1	Ras protein specific guanine nucleotide releasing factor 1	Stimulates the dissociation of GDP from RAS protein
ADAMTS9	ADAM metallopeptidase with thrombospondin type 1 motif 9	Implicated in the cleavage of proteoglycans, the control of organ shape during development, and the inhibition of angiogenesis
RhoB	Ras homolog family member B	Enable GTP binding activity; GTPase activity; and protein kinase binding activity.

Table S2. Selected down-regulated genes and its function in BRRF1 -overexpressed NPC43 cells.

Gene	Gene description	Gene function
RASA3	RAS p21 protein activator 3	Negative regulator of the Ras signaling pathway
RAG2	Recombination activating 2	Involved in the initiation of V(D)J recombination during B and T cell development
BTLA	B and T lymphocyte associated	Contains a single immunoglobulin (Ig) domain and is a receptor that relays inhibitory signals to suppress the immune response.
IL23R	Interleukin 23 receptor	Pairs with the receptor molecule IL12RB1/IL12Rbeta1, and both are required for IL23A signaling
GRIP2	Glutamate receptor interacting protein 2	Enable protein C-terminus binding activity
IL22RA2	Interleukin 22 receptor subunit alpha 2	The soluble protein specifically binds to and inhibits interleukin 22 activity by blocking the interaction of interleukin 22 with its cell surface receptor
GRID2	Glutamate ionotropic receptor delta type subunit 2	A member of the family of ionotropic glutamate receptors which are the predominant excitatory neurotransmitter receptors in the mammalian brain.
ABCB11	ATP binding cassette subfamily B member 11	A member of the superfamily of ATP-binding cassette (ABC) transporters
MAP3K7CL	MAP3K7 C-terminal like	Located on chromosome 21 and interact with GPS2

Table S3. Primers used for RT-qPCR

Gene	Forward primer 5'-3'	Reverse primer 5'-3'
GAPDH	ACAGTCCATGCCATCACTGCC	GCCTGCTTCACCACCTTCTTG
BTN3A1	TACACAACGTCACAGCCTCT	CTCCCTTGTTGTTGCTCCAC
BTN2A1	AGGAGTACCGAGGAAGAACCA	TGTGATGTTGTGTATGACCAGG
NLRC5	GCTCGGCAACAAGAACCTGT	GGTCCAAGGTCTCGTTCCT
BRRF1	CTGCTGGAAGACACCATCGT	AGGCATGGTGTCTGTCTTGG
RASGEF1A	GGACAGGGTCAGCAGCATTTA	CGGGTCCGGTGTTTCTTCTT
JAK3	CCTGATCGTGGTCCAGAGAG	GCAGGGATCTTGTGAAATGTCAT
AKR1D1	AGGTTGAGTGCCATCCGTATT	TCACCCAGATTGGATTCCTACTG
KRT31	TGTGGACCTGAATCGGGTG	CTGCGTGGTGAACCATTGC
SERPINA6	CTTCTATGTGGACGAGACAACTG	CCCACGTAGTTCATCTGCAC
GPR84	TTGGCATCTTCTATTGCCTCATC	TGTCGCAACTTGTATTGGTCC
IKBKGP1	CAGAGCCTGGCATTCCTTAGT	TGCTGACAGGAAGTGGCTTTT
DUSP9	TTCCGCCAATTTGGAGAGCC	TGCTTGTAGTGAAAGTCACCATT
RASGRF1	TACTCGGCCATGTCACCCTT	GGGTCGTATCGCCCTCATC
ADAMTS9	CAACCCCTGGATGTGGTCAAA	AGTTGGACAGGCAAAGGGTAG
RHOB	ATCCCCGAGAAGTGGGTCC	CGAGGTAGTCGTAGGCTTGGA
RASA3	AATGGGCAATGTGACCCCTAC	CGAACTGGGGATTGTTGGTCTT
RAG2	GGACGCTCATACATGCCTTCT	GGCAAGTGAATGTCCTCCTAAA
BTLA	CATCTTAGCAGGAGATCCCTTTG	GACCCATTGTCATTAGGAAGCA
IL23R	ACATGCTTCTATGTACTGCACTG	TGTGTCTATGTAGGTGAGCTTCC
GRIP2	GGCTGCTCAGTGTCGATGG	CATTAGCCACCGTGTCAGG
IL22RA2	TGTTGGGGTACTCAAGAACTCT	CCCTCCCGTAATAAGGTTCCTG
GRID2	TGATGAGGTATTTCGCACTGC	GGCCAAGATGCCTTGATTCATAA
ABCB11	TGACTACGACGTTGAGTTACAAG	CTGCGGCAATGACCCAAAA
MAP3K7CL	GTGCGGGGTCATTTGTCTCA	GGATTTCCACGGGCACAGTA
JAK1	CCACTACCGGATGAGGTTCTA	GGGTCTCGAATAGGAGCCAG
JAK2	ATCCACCCAACCATGTCTTCC	ATTCCATGCCGATAGGCTCTG
TYK2	GGAGGAGGGTTCTAGTGGCA	ATGTCCCGGAAGTCACAGAAG
IL22	GCTTGACAAGTCCAACTTCCA	GCTCACTCATACTGACTCCGT
ICAM1	ATGCCCAGACATCTGTGTCC	GGGGTCTCTATGCCCAACAA
RAGE	GTGTCCTTCCCAACGGCTC	ATTGCCTGGCACCGGAAAA
ULBP-3	TCTATGGGTCACCTAGAAGAGC	TCCACTGGGTGTGAAATCCTC
ULBP-4	GCACTTGGGGAGAATTGACCC	ACATCTCGACTTGCAGAGTGG

Table S4. Antibodies used in this study.

Antibody against	Clone	Cat. No.	Purchased from
BTN2A1	polyclonal	orb499606	Biorbyt
BTN3A1	7F4F1	ABIN5684130	antibodies-online.com
NLRC5	3H8	MABF260	Sigma
Myc tag		60003-2-Ig	Proteintech
p-STAT5 (Tyr694)	D47E7	4322	Cell Signaling Technology
STAT5a	51	Sc-136081	Santa Cruz Biotechnology
SAPK/JNK	polyclonal	9252	Cell Signaling Technology
p-SAPK/JNK	81E11	4668	Cell Signaling Technology
STAT1	1F7C6	66545-1-Ig	Proteintech
p-STAT1 (Tyr701)	58D6	88845	Cell Signaling Technology
β-Actin	8H10D10	3700T	Cell Signaling Technology
GAPDH	D16H11	5174	Cell Signaling Technology
STAT3	124H6	9139	Cell Signaling Technology
p-STAT3 (Tyr705)	3E2	9138	Cell Signaling Technology
HRP donkey anti-rabbit	__________	AP182P	Merck Millipore
HRP goat anti-mouse	__________	AP130P	Merck Millipore
HRP goat anti-rat	__________	AP131P	Merck Millipore
Jak1	6G4	3344	Cell Signaling Technology
p-Jak1(Tyr1034/1035)	D7N4Z	74129	Cell Signaling Technology
Jak2	D2E12	3230	Cell Signaling Technology
p-Jak2 (Tyr1008)	D4A8	8082	Cell Signaling Technology
Jak3	D1H3	8827	Cell Signaling Technology
p-Jak3 (Tyr980/981)	D44E3	5031	Cell Signaling Technology
Tyk2	D4I5T	14193	Cell Signaling Technology
p-Tyk2 (Tyr1054/1055)	D7T8A	68790	Cell Signaling Technology
